# Tension of knotted surgical sutures shows tissue specific rapid loss in a rodent model

**DOI:** 10.1186/1471-2482-11-36

**Published:** 2011-12-21

**Authors:** Christian D Klink, Marcel Binnebösel, Hamid P Alizai, Andreas Lambertz, Klaus T vonTrotha, Elmar Junker, Catherine Disselhorst-Klug, Ulf P Neumann, Uwe Klinge

**Affiliations:** 1 Department of Surgery, RWTH Aachen, Germany; 2 Applied Medical Engineering, Helmholtz Institute, RWTH Aachen, Germany

**Keywords:** suture tension, cutting reaction, collagen, suture material, polypropylene, tension sensor

## Abstract

**Background:**

Every surgical suture compresses the enclosed tissue with a tension that depends from the knotting force and the resistance of the tissue. The aim of this study was to identify the dynamic change of applied suture tension with regard to the tissue specific cutting reaction.

**Methods:**

In rabbits we placed single polypropylene sutures (3/0) in skin, muscle, liver, stomach and small intestine. Six measurements for each single organ were determined by tension sensors for 60 minutes. We collected tissue specimens to analyse the connective tissue stability by measuring the collagen/protein content.

**Results:**

We identified three phases in the process of suture loosening. The initial rapid loss of the first phase lasts only one minute. It can be regarded as cutting through damage of the tissue. The percentage of lost tension is closely related to the collagen content of the tissue (r = -0.424; p = 0.016). The second phase is characterized by a slower decrease of suture tension, reflecting a tissue specific plastic deformation. Phase 3 is characterized by a plateau representing the remaining structural stability of the tissue. The ratio of remaining tension to initial tension of phase 1 is closely related to the collagen content of the tissue (r = 0.392; p = 0.026).

**Conclusions:**

Knotted non-elastic monofilament sutures rapidly loose tension. The initial phase of high tension may be narrowed by reduction of the surgeons' initial force of the sutures' elasticity to those of the tissue. Further studies have to confirm, whether reduced tissue compression and less local damage permits improved wound healing.

## Background

Surgery needs tissue approximation, which is achieved by sutures, for every tissue in a widely standardised manner [1]. Failure of surgical sutures leads to relevant complications after surgical interventions [2]. Amongst others burst abdomen after fascial closure [3] and anastomotic leakage after intestinal anastomosis [4] have to be mentioned. Dissection and extent of local devascularisation is known to be important, because it reduces the blood supply [5]. Furthermore, several experimental studies had demonstrated, that any high suture tension additionally has a negative influence on the quality of wound healing by inducing ischemia, oedema and tissue necrosis [6-8]. Despite, even today we are not able to control the tensile strength that is applied when knotting surgical sutures [9,10]. A correctly performed suture usually is related vaguely to "clinical experience" of the surgeon [9,10]. Mainly, a complete cutting off (= too high tension) [11] or a dehiscence of the tissue (= too little tension) [2] indicates a wrong technique. It is assumed, that between these 2 extremes the tensile strength is widely constant and differences are of no major importance.

Thus, in the present study we investigated whether the tension within a suture loop is of considerable variation within the first hour after application of the knot, and whether there are considerable differences between various tissues. Therefore we measured the tension within a single monofilament suture of size 3/0 polypropylene at 5 different tissues in a rabbit model continuously for 60 minutes.

## Methods

The experiments were officially approved by the Animal Care and Use Review Committee of the Russian State Medical University, Moscow, Russia and are conformed to the Helsinki Declaration. All animals received humane care in accordance with the requirements of the German Tierschutzgesetz, §8 Abs. 1 and in accordance to the *Guide for the Care and Use of Laboratory Animals *published by the National Institute of Health. All animals were kept under standardized conditions: temperature between 22°C and 24°C; relative humidity 50-60%; 12 h of light following 12 h of darkness. The animals had free access to food and water. Food was withdrawn 12 h before and after surgery.

**Figure 1:**
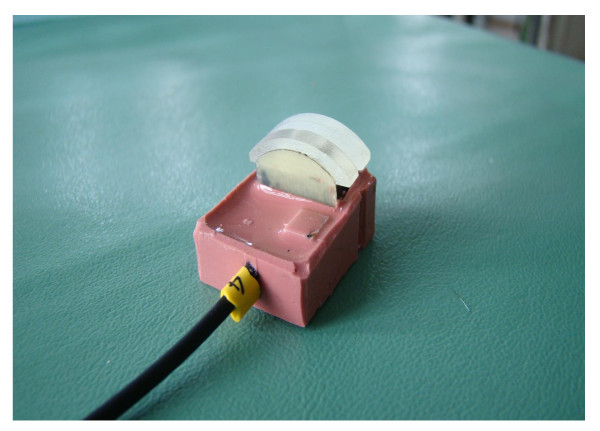
**Used forced sensor for tension measurements developed by Applied Medical Engineering, Helmholtz Institute, RWTH Aachen, Germany**.

**Figure 2:**
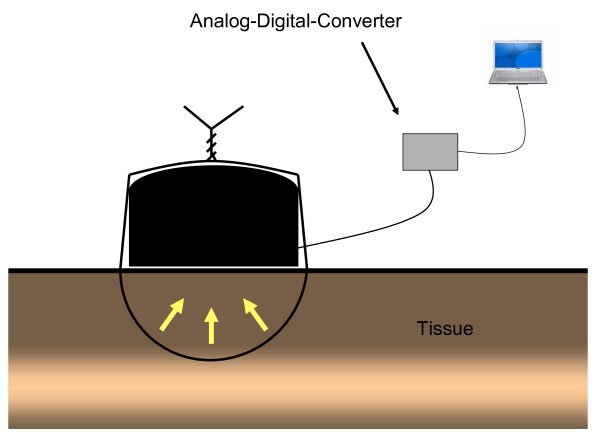
**Model of experiment settings showing placed suture upon sensor and involved tissue**. Yellow arrows are indicating force applied to tissue by knotting.

In 3 female rabbits with a mean bodyweight of 3500 g 3/0 monofilament polypropylene single sutures (Prolene^®^) were placed in skin, muscle, liver, stomach and small intestine. In total up to seven separate single sutures were placed in each organ receiving 6 measurements for each organ without specific consecutive order of placement. Measurements were performed twice in each animal. Dynamic of suture tension was documented in each suture for 60 minutes. The suture tension was measured by a customised force sensor, which was developed by the Institute of Applied Medical Engineering, RWTH Aachen University, Germany. The patent application of the force sensor is still in progress. The analogue force data have been digitised by a 16 bit A/D converter and stored on a PC with a sampling frequency of 250 Hz. The resolution of the measurement set-up was 0,077g and due to the mechanical set up (Figure 1 and 2) the measured force was proportional to the suture tension. After measuring force data have been processed with the signal acquisition toolbox of Matlab^® ^MathWorks.

### Surgical procedure

**Figure 3:**
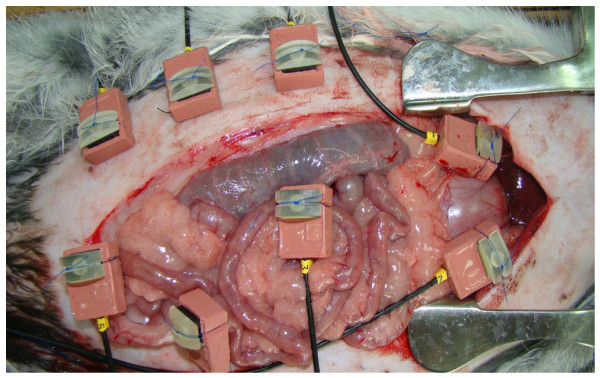
**Sensors placed on skin (3 sensors), stomach (2 sensors) and small intestine (3 sensors) during documentation**.

Surgical procedure was done at the Joint Institute for Surgical Research of the Russian State Medical University, Moscow, Russia. After induction by isoflurane, general anesthesia was achieved with a subcutaneous mixture of 0.3 mg/kg medetomidine and ketamine hydrochloride 100 mg/kg. The rabbits were weighed, and their skin was shaved and disinfected with polyvidone-iodine solution. The animals were fixed in a supine position. A standardized 15 cm median laparotomy was performed. Suture material was placed by puncture in the tissue with a 1/2 circle curved needle. Distance between penetration points was 1 cm independently of the sutured tissue. After placement of the sensor the suture was tied by four standardized single knots by hand-tied method. All knots were sutured by the corresponding author by a right-hand technique placing 3 knots in the same direction and the last one in the opposite direction. Suture tension was documented in real time and was digitally transferred to a personal computer for period of 60 minutes (Figure 3). The animals were sacrificed after measurements were done. At time of explantation tissue specimens of the different organs were removed for histological and immunohistochemical investigations.

### Collagen/Protein content

All the tissue sections used were obtained 3 mm thick each. They were cut with a razor blade and immediately fixed in 10% formalin in 0. 1 M phosphate buffer, pH 7.2 containing 0.15 M NaCI. Samples were embedded in paraffin and sections, approximately 10 µm thick, were obtained. They were placed in small test tubes (10 × 75 mm). Groups of 5 sections were deparaffinised after incubation with xylol, xylol: ethanol (1 : 1), ethanol, water : ethanol (1 : 1), and water. Subsequently sections were covered with 0.2 ml of a saturated solution of picric acid in distilled water that contained 0.1% Fast green FCF and 0.1% Sirius red. The tubes were covered with aluminium foil and incubated at room temperature for 30 mm on a rotary shaker. Fluids were carefully withdrawn with a disposable pipette and the sections were rinsed several times with distilled water until the fluid was colourless. One millilitre of 0.1 N NaOH in absolute methanol (1 : 1, v : v) was then added and each tube gently mixed until all the colour was eluted from the section (usually within a few seconds). The eluted colour was read immediately in a Beckman 35 spectrophotometer at 535 and 605 nm, i.e., the wavelengths corresponding to the maximal absorbance of Sirius red F3BA and Fast green FCF, respectively. The sections were saved for collagen and protein estimations, vide infra.

### Fitted model for calculation of Constant Declining Phase

**Figure 4:**
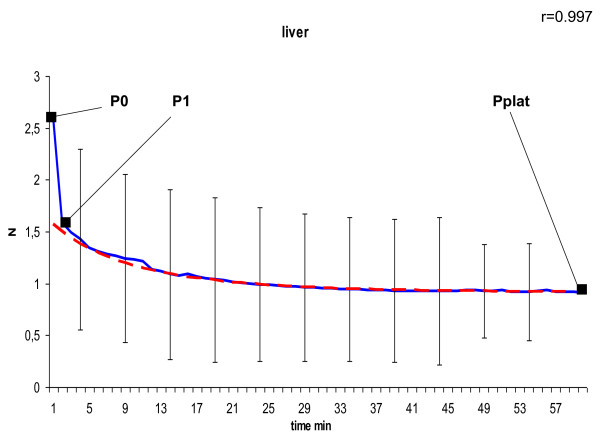
**Model of dynamic of suture tension in liver showing measured tension strength within the suture loop (solid blue), and estimate (fitted model according to formula above; solid red) for the course of tension between P_1 _and P_plat_, assuming a final plateau and a constant rate for declination; relation of the fitted model with the measured curve is indicated by Pearson's correlation coefficient r**. P_0 _= peak suture tension, P_1 _= transition to the constant declining phase, P_plat _= suture tension of the constant plateau (mean ± SD).

**Figure 5:**
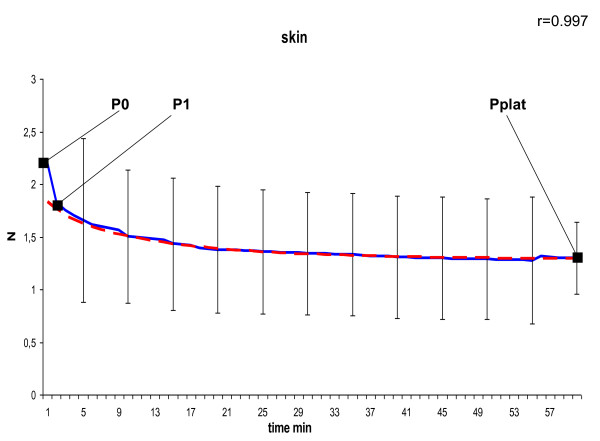
**Model of dynamic of suture tension in skin showing measured tension strength within the suture loop (solid blue), and estimate (fitted model according to formula above; solid red) for the course of tension between P_1 _and P_plat_, assuming a final plateau and a constant rate for declination; relation of the fitted model with the measured curve is indicated by Pearson's correlation coefficient r**. P_0 _= peak suture tension, P_1 _= transition to the constant declining phase, P_plat _= suture tension of the constant plateau (mean ± SD).

**Figure 6:**
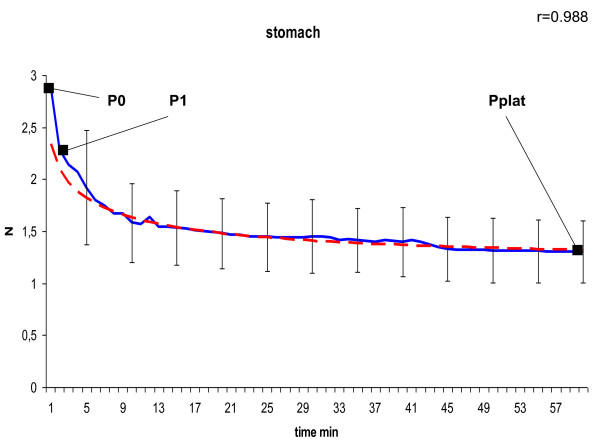
**Model of dynamic of suture tension in stomach showing measured tension strength within the suture loop (solid blue), and estimate (fitted model according to formula above; solid red) for the course of tension between P_1 _and P_plat_, assuming a final plateau and a constant rate for declination; relation of the fitted model with the measured curve is indicated by Pearson's correlation coefficient r**. P_0 _= peak suture tension, P_1 _= transition to the constant declining phase, P_plat _= suture tension of the constant plateau (mean ± SD).

**Figure 7:**
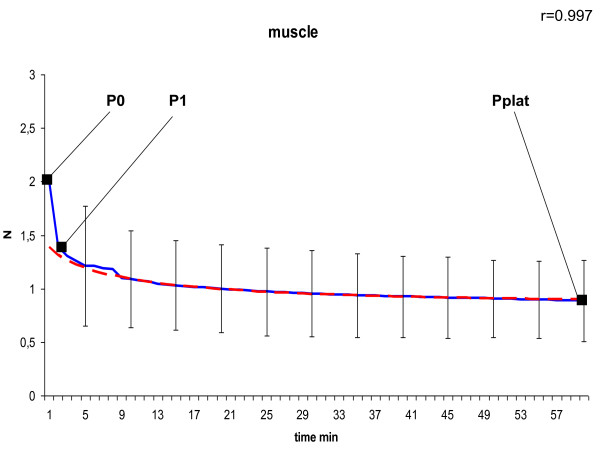
**Model of dynamic of suture tension in muscle showing measured tension strength within the suture loop (solid blue), and estimate (fitted model according to formula above; solid red) for the course of tension between P_1 _and P_plat_, assuming a final plateau and a constant rate for declination; relation of the fitted model with the measured curve is indicated by Pearson's correlation coefficient r**. P_0 _= peak suture tension, P_1 _= transition to the constant declining phase, P_plat _= suture tension of the constant plateau (mean ± SD).

**Figure 8:**
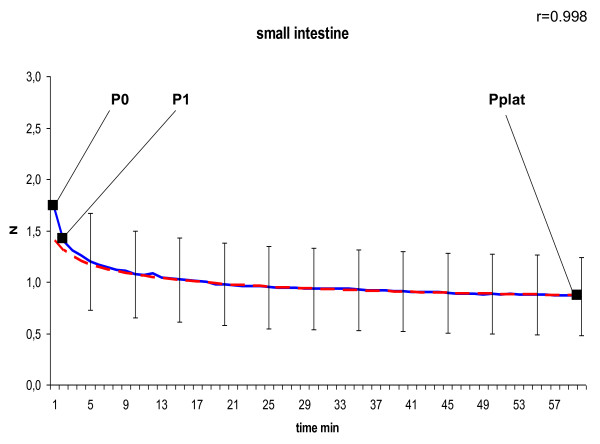
**Model of dynamic of suture tension in small intestine showing measured tension strength within the suture loop (solid blue), and estimate (fitted model according to formula above; solid red) for the course of tension between P_1 _and P_plat_, assuming a final plateau and a constant rate for declination; relation of the fitted model with the measured curve is indicated by Pearson's correlation coefficient r**. P_0 _= peak suture tension, P_1 _= transition to the constant declining phase, P_plat _= suture tension of the constant plateau (mean ± SD).

In the curve of the measured tension we defined P_0 _as peak tension at the beginning, P_1 _as tension after 1 minute at the transition point to slow decrease due to a negative constant gradient, and P_plat _as tension of the final plateau after 60 minutes. To model the declining course of the suture tension of the second phase shown in Figure 4, 5, 6, 7 and 8 we used the following formula:

$$y=a*exp\left(-b*t^{c}\right)+d$$

a = Estimated P_1 _- P_plat_

b = Estimated negative gradient

c = Estimated time gradient

d = Estimated P_plat_

### Statistical analysis

Statistical analysis has been carried out using the Statistical Package for Social Sciences software (SPSS^®^, Vers.17.0). Differences of the tension were analyzed by Kruskal-Wallis test for non-parametric data and in case of significant differences confirmed by Mann-Whitney test. Pearson's correlation coefficient reflects functional relationship between numeric data. P-values < 0.05 were considered to be significant. All data are represented as mean ± standard deviation.

## Results

In general, in all measurements the course of suture tension similarly showed an exponential decrease at the beginning, ending up in a plateau (Figure 4, 5, 6, 7, 8). In order to grasp the dynamic of suture tension we decided to analyse three periods separately: phase 1 = rapid cutting phase RCP (time period between P0 and P1; 1 minute), phase 2 = constant declining phase CDP (P1 until P2; 60 minutes), phase 3 = plateau phase PP (time period after P2; > 60 minutes).

Overall, to reduce the tension to half of the initial peak tension it took only 24 ± 21 min in the liver and 35 ± 24 min in the muscle. In stomach, in small intestine and in skin half of the tension was not reached yet after 60 min (Table 1).

**Table 1:** Characteristics of suture tension depending on the involved tissue

	Liver (n = 6)	Skin (n = 6)	Stomach (n = 6)	Muscle (n = 6)	Small intestine (n = 6)
P_0 _in N	2.6 ± 1.6	2.2 ± 1.0	2.9 ± 1.8	2.0 ± 0.7	1.7 ± 0.6

P_1 _in N	1.6 ± 1.1	1.8 ± 0.9	2.3 ± 1.1	1.4 ± 0.6	1.4 ± 0.5

P_plat _in N	0.9 ± 0.7	1.3 ± 0.6	1.3 ± 0.3	0.9 ± 0.4	0.9 ± 0.4

Decrease of tension in the RCP in %	34 ± 19	16 ± 10	18 ± 10	27 ± 17	17 ± 7

Estimated decrease of tension in the CDP per minute in %	8	8	15	15	10

Relation of P_plat _to P_0 _in %	40 ± 18	60 ± 9	55 ± 19	46 ± 9	51 ± 11

Time after half of the tension is reached in min	24 ± 21	> 60	> 60	35 ± 24	> 60

Collagen/Protein Content in µg/mg	25 ± 4	45 ± 11	49 ± 14	43 ± 4	44 ± 8

### Rapid Cutting Phase

Though we tried to place the sutures with the same tension there still was a considerable variation in a range from 0.7 to 5.9 N.

Initial peak tension of stomach sutures was highest with a mean of 2.9 ± 1.8 N, followed by those of liver sutures with a mean of 2.6 ± 1.6 N. The mean initial tension of skin sutures was 2.2 ± 1.0 N. Mean initial tension of muscle sutures was slightly lower with 2.0 ± 0.7 N, whereas mean initial tension of thin bowel sutures was lowest with 1.7 ± 0.6 N. However, due to the considerable variations there were no significant differences between the groups.

The initial loss of suture tension within the first minute is highest in liver with 34 ± 19%, 27 ± 17% in muscle, 18 ± 10% in stomach, 17 ± 7% in small bowel, and lowest in skin sutures with 16 ± 10%. The loss of suture tension in liver sutures was significantly higher than in skin sutures (p = 0.014). Comparisons of other tissues did not show any significant differences.

### Constant Declining Phase

The constant phase is characterised by a loss of suture tension with a negative gradient of constantly 8 - 10% per minute in liver, skin or small bowel, and 15% per minute in both stomach and muscle. Remodelling of the curves with these negative gradients showed widely similar courses with correlation coefficients of r > 0.95 (table 1) between the estimated and the measured tension, confirming the constancy of the declining speed.

### Plateau Phase

After 60 minutes a plateau phase was reached when the decrease of suture tension tends towards zero. This plateau was quite uniformly either at 0.9 ± 0.7 N in liver sutures, 0.9 ± 0.4 N in muscle sutures and 0.9 ± 0.4 N in small intestine sutures, or at 1.3 ± 0.6 N in skin sutures, and 1.3 ± 0.3 N in stomach sutures (table 1). The remaining plateau of stomach sutures was significantly higher than in muscle sutures (p = 0.042) and small intestine sutures (p = 0.045).

### Collagen/Protein Content

The Collagen/Protein Content was lowest in liver with 25 ± 4 µg/mg, followed by 43 ± 4 µg/mg in muscle, 44 ± 8 µg/mg in small intestine, 45 ± 11 µg/mg in skin, and 49 ± 14 µg/mg in stomach. The Collagen/Protein Content had a negative correlation with the loss of suture tension in phase 1 (r = - 0.424; p = 0.016), meaning less collagen is linked to higher percentage of rapid tension loss. Furthermore, the Collagen/Protein Content had a positive correlation with the percentage of the plateau in relation to the initially applied tension (r = 0.392; p = 0.026), indicating higher amount of collagens in tissues with higher plateau tension, and consecutively higher stability.

## Discussion

Despite considerable improvements in surgery, the incidence of failure of surgical sutures, remained widely constant throughout the last decades [12]. The negative influence of high suture tension on the structural and mechanical quality of the healing incision has been clearly demonstrated by several authors, focussing on parameters like suture material, suture technique and the suture-length to wound-length ratio [7,8,13]. Nevertheless, as several meta-analysis outlined we seem to be unable to substantially reduce our rate of anastomotic leakage or of incisional hernia by changing surgical technique [14-16]. However, the disappointing lack of improved results by optimising suture material or suture technique may find its explanation in the negligence of suture tension in most of the experimental settings. Until now surgeons have no other criteria than their purely subjective 'feeling' of what the tissue needs in terms of suture tension and the local tissue damage. In regard to the limited visual control in the process of suturing, the surgeon has to rely on his firm belief that his suture technique and suture tension are 'appropriate' for the tissue. Repeated measurements of the suture tension, 5 sutures subsequently done by one surgeon, demonstrates a considerable variation between these sutures [9]. Furthermore, the range of the tension considered as "appropriate" showed wide overlap to too high or too loose tension. Because of missing data, which define the optimum of a suture tension, surgical suture repair is mainly based on an individual feeling for suture tension.

The tension within a suture loop will be affected by the volume (bite) and type of tissue included, the size and diameter of the suture, and the force applied during knotting. A rabbit model was the most suitable model for our setting since the sensors are too big for a rat model. In our study we used only three rabbits but not the amount of animals is important but rather the amount of measurements per tissue in order to obtain reliable data. We used 3/0 monofilament polypropylene single sutures which is an established standard suture material. Although we tried to place similar sutures with similar bite and tension there still was a considerable variation of 0.7 to 5.9 N for the peak tension. Obviously, we were unable to apply constant peak tension to a knot, which is a clear limitation of our study but in accordance with findings of Butz et al. [9]. Obviously not only between surgeons it seems to be impossible to standardize suture tension in hand-knotted sutures [10] but also the same surgeon underlies a great variability of suture tension since all sutures were performed by the same person. Therefore, it might be favourable to develop a suture device which provides standardized suture tension. However, we usually saw a rapid decrease within the first minute, which was interpreted as initial cutting through. This loss was higher in case of high peak tension, but was affected as well by the resistance of the tissue. A high amount of collagen seems to withstand better to the forces and thus reduce the cutting through. It may be speculated that the extent of cutting through damage of the tissues impairs wound healing and favours failures e.g. incisional hernia or anastomotic leakage. Adoption of the peak tension within the suture to the tissue should reduce the amount of damage, and is that what the experienced surgeon is able to consider already today reducing this surplus to a minimum for achieving less necrosis and improved wound healing.

The constant decline of the tension during what we call Phase II is characterized by a constant loss of suture tension of 8 - 15% per minute. This can be interpreted as plastic deformation and was slightly different between the tissues. The differences may be caused by distinct composition of the tissues and their ECM, however it could not been related to the collagens or any other biometric variable. Further measurements will show whether this phase and its area under the curve indicating time with increased tension may be reducible with stretchable sutures.

With all sutures we could see a rapid loss of tension, though sometimes it takes more than 1 hour to reduce the initial peak tension to half. The strength of the remaining plateau tension mainly depended on the tissue, and furthermore, was closely related to the amount of collagen. The collagen per protein content was found to have a negative correlation to the decrease of tension in the rapid cutting phase RCP. This fact indicates that especially in tissue with low collagen content (liver) high tension can lead to overwhelming tissue damage whereas in tissue of high overall collagen content (stomach, skin) this cutting reaction is not as severe since collagen is one of the structural proteins that is responsible for the tissues' stability [17,18]. There are many other factors supposedly effecting the stability of tissue, like elastin [19], chondroitin sulphate [20], the composition of the various collagens and its cross-linkings, [21-23], the junction between the cells [24], or adhesion molecules [25]. All this may influence the resistance of tissue, its plastic deformation or its resident plateau, and should be considered in further experiments.

The design of our device allowed only the evaluation of single sutures so far. Although with running sutures a more even distribution of suture tension along the incision is attained and tension peaks are avoided, the question of adequate suture tension remains unanswered. It is technically not easy to maintain identical tension levels from stitch to stitch in a running suture. This might lead to a generally lower suture tension in running sutures compared to single sutures, which can be a possible explanation for the superior quality of fascial healing after running sutures [26]. We are working on an experimental layout of our sensor in order to investigate tension of running sutures. Furthermore, the investigation of elastic fibres might highlight important findings of tension deviation.

With knowledge of the influence of inadequate suture tension on the healing of laparotomy wounds, further research work needs to focus on the definition of a tissue specific optimum for suture tension, and the development of sutures and measurement devices which help the surgeon to suture according to this tension optimum.

## Conclusion

Knotted non-elastic monofilament sutures rapidly loose tension independently of the sutured tissue. The initial phase of high tension might be influenced by the surgeons' initial force to the sutures. Further studies have to confirm, whether reduced tissue compression leads to less local tissue damage and therefore permits improved wound healing.

## Competing interests

The authors declare that they have no competing interests.

## Authors' contributions

CDK and MB have made substantial contributions to conception and design. MB and AL have been involved in revising the manuscript critically for important intellectual content. HPA, AL and KTT contributed to acquisition of data. CDK has been performing the statistical analysis. CD and UK have been involved in analysis and interpretation of data. EJ has been involved in design and develop the sensor and in interpretation of data. UPN has given final approval of the version to be published. All authors read and approved the final manuscript.

## Pre-publication history

The pre-publication history for this paper can be accessed here:

http://www.biomedcentral.com/1471-2482/11/36/prepub
